# Supplementary material for: The Estrogen Receptor α Signaling Pathway Controls Alternative Splicing in the Absence of Ligands in Breast Cancer Cells
Source: Cancers (Basel). 2021 Dec 13;13(24):6261. doi: 10.3390/cancers13246261 (PMC8699117; doi:10.3390/cancers13246261)
Supplement: Supplementary file 1 [file cancers-13-06261-s001.zip › Supplementary_Figures.pdf]

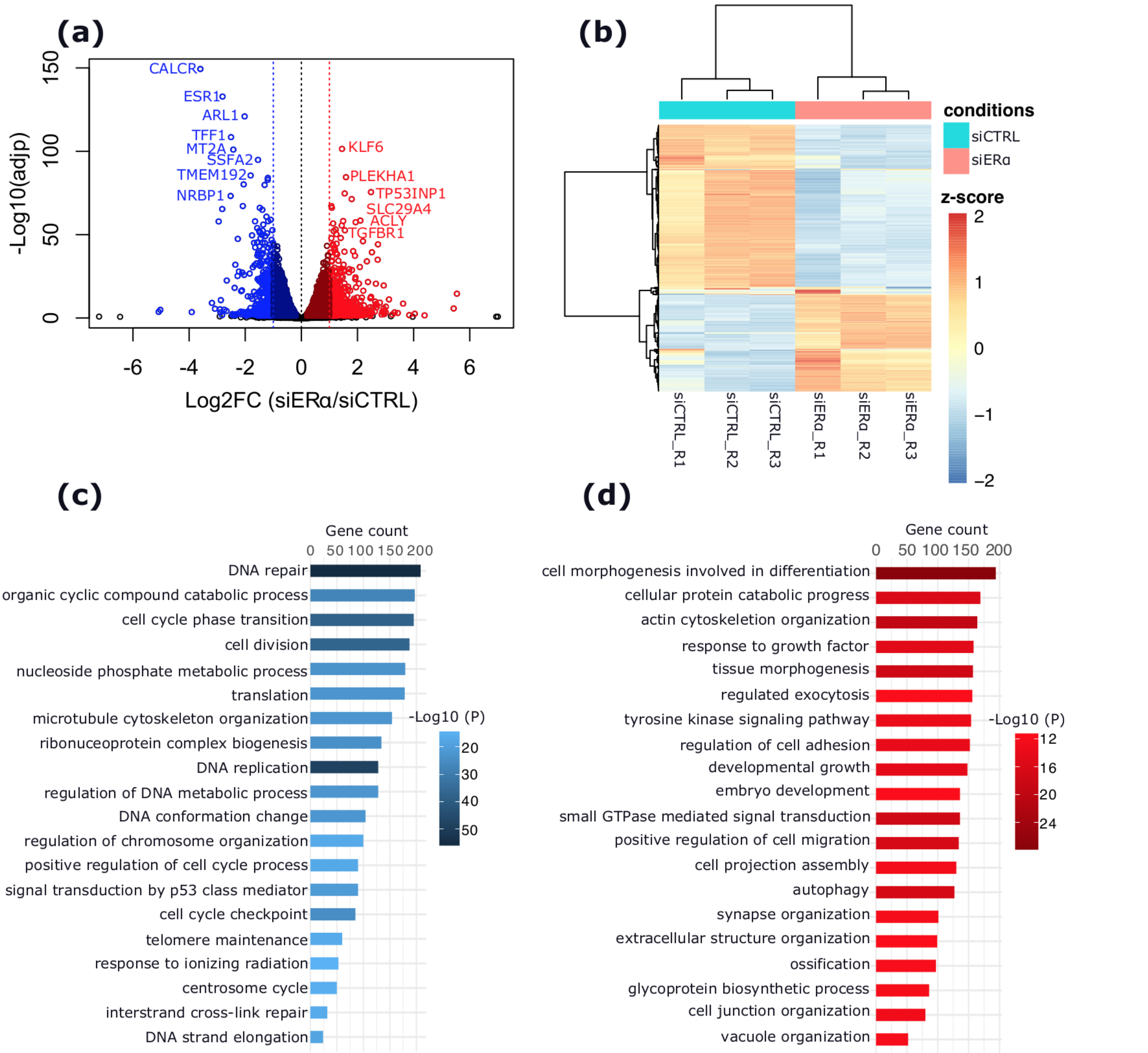

Figure S1. The transcriptional effects of apoERα depletion in hormone-starved MCF-7 BC cells. **(a)** Volcano plot Scheme 2. FC and significance (adj-p) of genes upon apoERα silencing. In blue color are represented downregulated genes while in red are represented upregulated genes. **(b)** Heat map plot showing the top 500 changing genes upon ERα gene silencing. Color intensities correspond to z-score calculated as a difference between mean and variance over samples. Negative and positive z-scores correspond to downregulated and upregulated genes, respectively. **(c-d)** Bar plots showing the gene ontology (GO) enrichment analysis related to downregulated and upregulated genes, respectively. Bar size represents the number of genes overlapping each enriched GO term and color intensities are proportional to the significance (*p*-value) of the enriched GO terms.

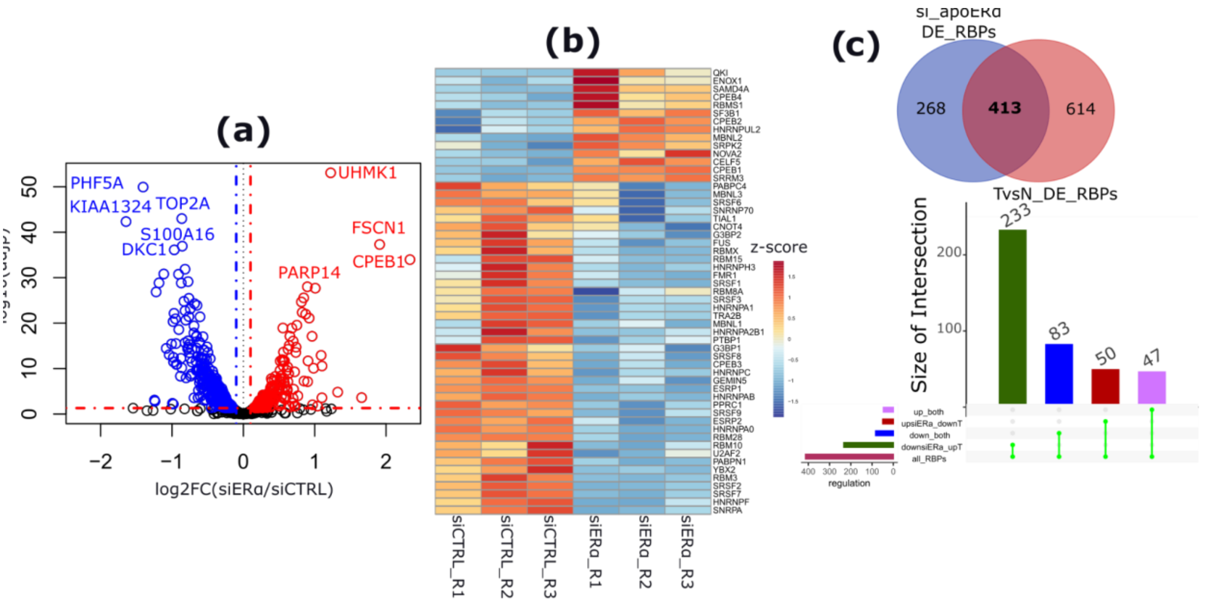

Figure S2. gene expression changes in apoERα-regulated RBPs. **(a)** Volcano plot showing the log2FC and significance (adj-p) of the apoERα-regulated RBPs. apoERα-induced and apoERα-repressed RBPs are shown in blue and red colors, respectively. **(b)** Heat map plot comparing the expression levels (in TPM units) of apoERα-regulated SFs. Negative and positive z-scores correspond to downregulated and upregulated SFs, respectively. **(c)**, Venn diagram reporting the number of apoERa-regulated RBPs that were found as DE comparing breast tumor versus adjacent normal samples. Upset plot reporting the coherence of regulation of the overlapping (413) RBPs shown in (c).

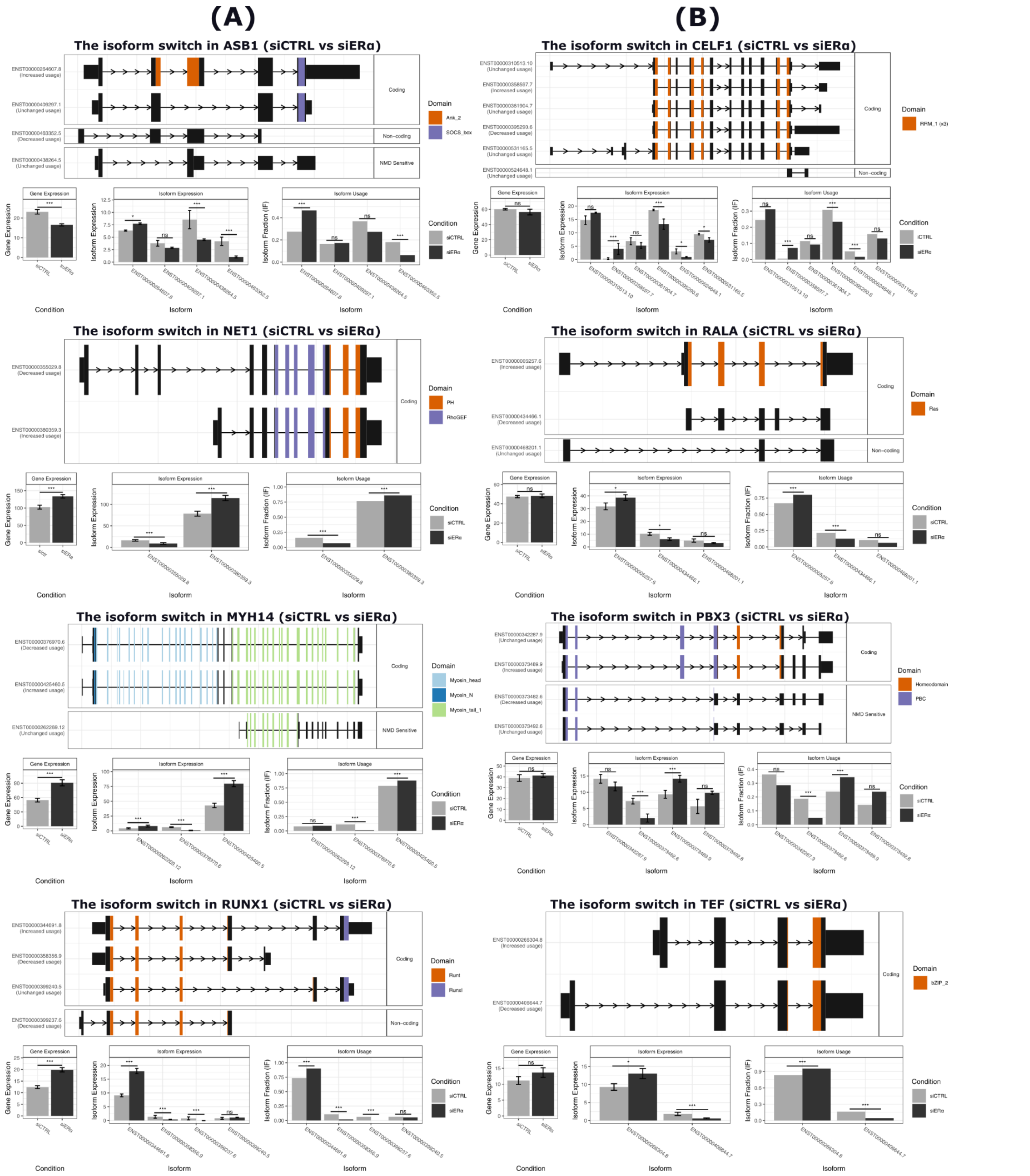

Figure S3. Selected example genes with switching isoform pairs upon apoERα silencing in MCF-7 cells. **(A)** examples of apoERα-regulated genes with isoforms responding in opposite directions. **(B)** examples of genes not regulated by apoERα at gene level, but regulated at the isoform level only.

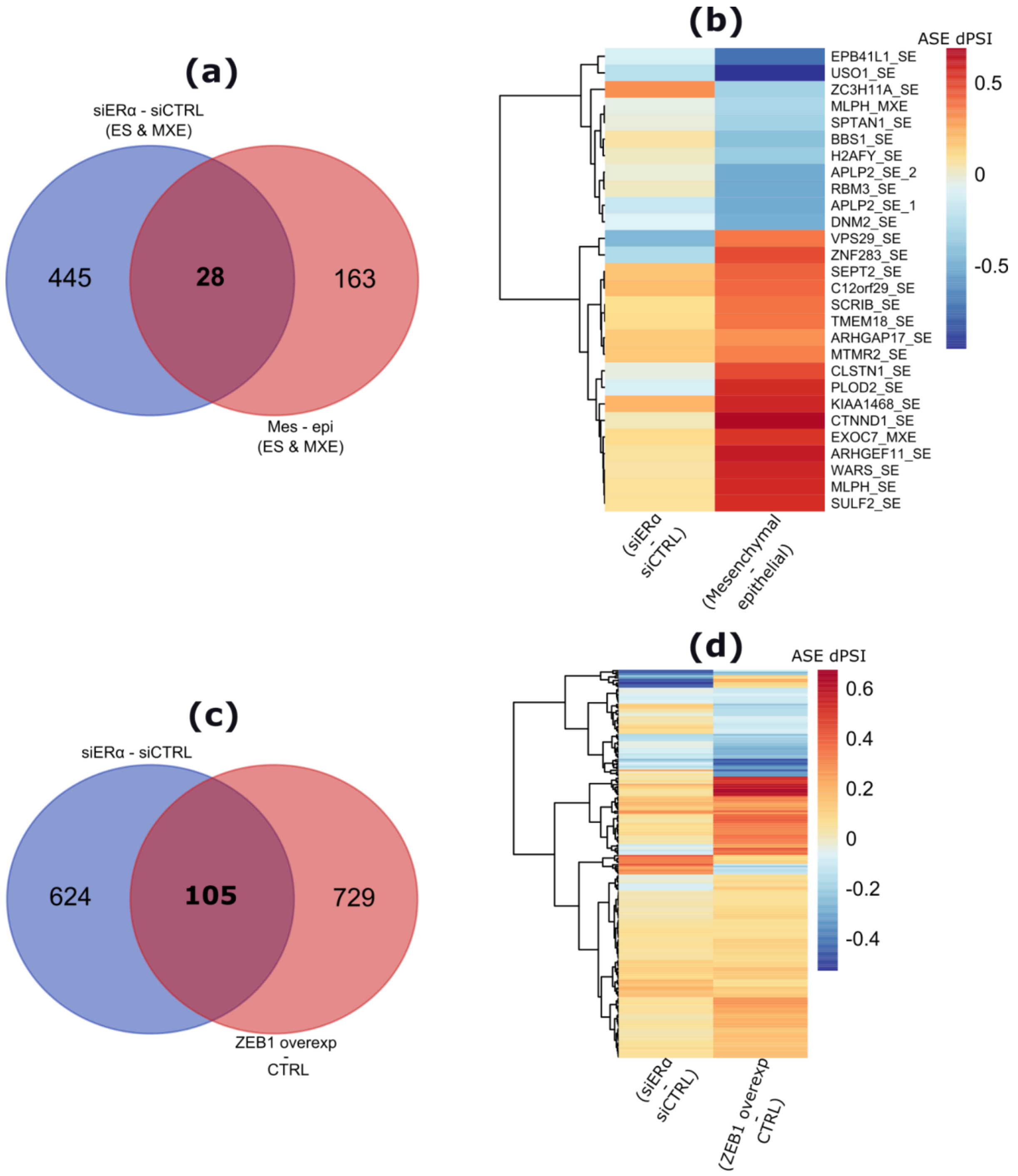

Figure S4. Overlapping ASEs regulated by apoERα silencing in this study with that identified as differentially regulated between (i) epithelial and mesenchymal BC cell lines (GSE30290) **(a,b)** [1], and (ii) upon ZEB1 overexpression in H358 epithelial cells (GSE75492) **(c,d)** [2], respectively. In GSE30290 only ES and MXE events were publically available. For each comparison on the left is reported the number of overlapped ASEs while on the right the heatmaps report the dPSI of the overlapped events.

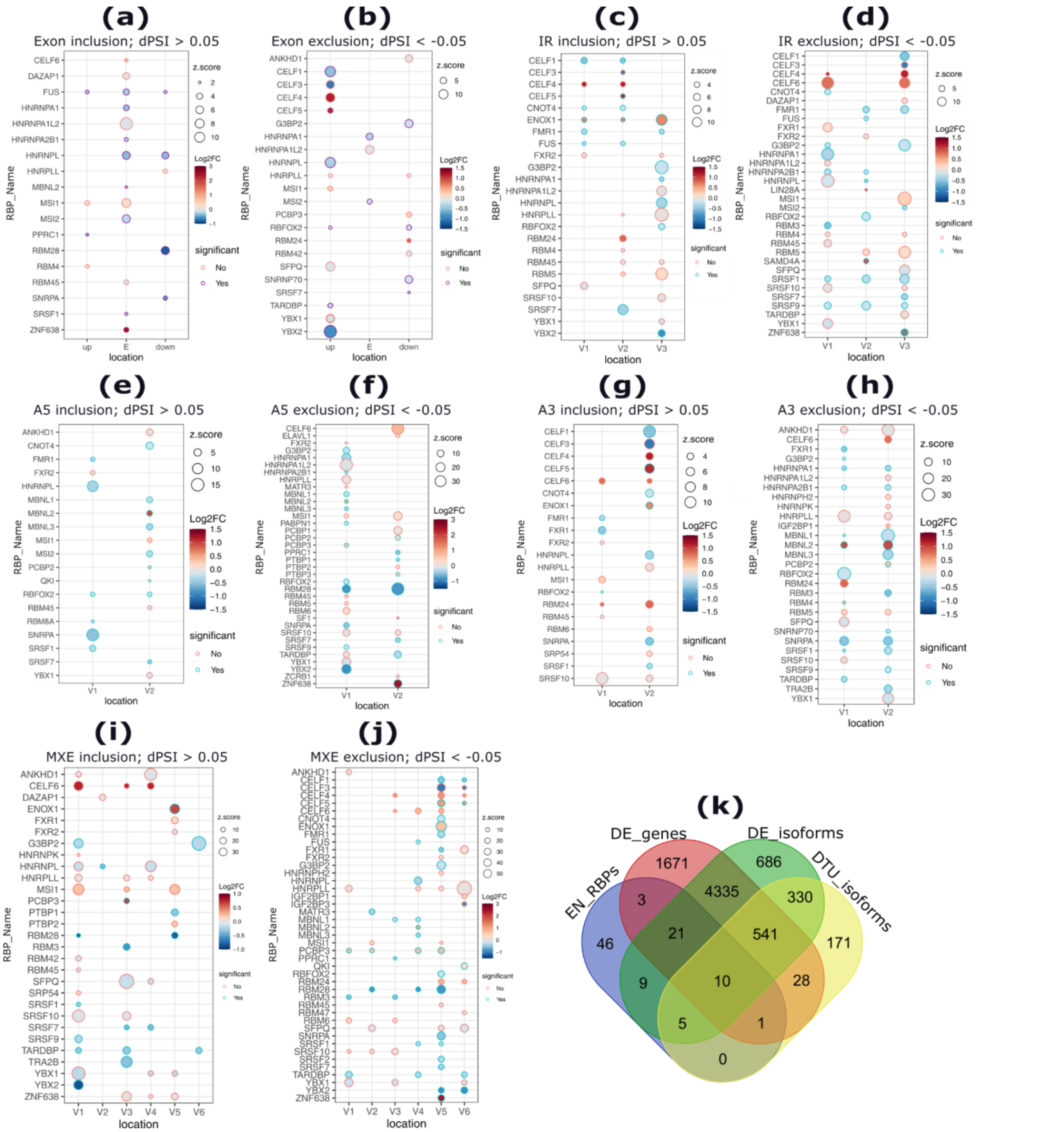

Figure S5. Overview of the RBP-binding motifs enrichment analysis performed on ASEs reported upon apoERα depletion in MCF-7 cells. **(a–j)** dot plots reporting the enriched RBP motifs for ES, RI, A5′SS, A3′SS, and MXE events, respectively. The name of enriched RBP is provided on the y-axis of the plot. The x-axis represents the positions where the binding motif is predicted to be enriched (up: upstream intron; E: exon, down: downstream intron). The color intensities represent the DE status of enriched RBPs (red for upregulated and blue for downregulated). The significance of the DE status of the RBP is represented by the border color of the dot. The size of the dot is proportional to the enrichment z-score (sig: z > 1.96). (V1 to V3) in **(c,d)** represent upstream, within the intron, or downstream exon, respectively. (V1 to V6) refer to genomic regions upstream, within and downstream of the first (V1 to V3) and second (V4 to V6) exons involved in the MX event, respectively. **(k)** Venn Diagram representing the number of enriched RBPs and whether they are or not regulated at gene or isoform level.

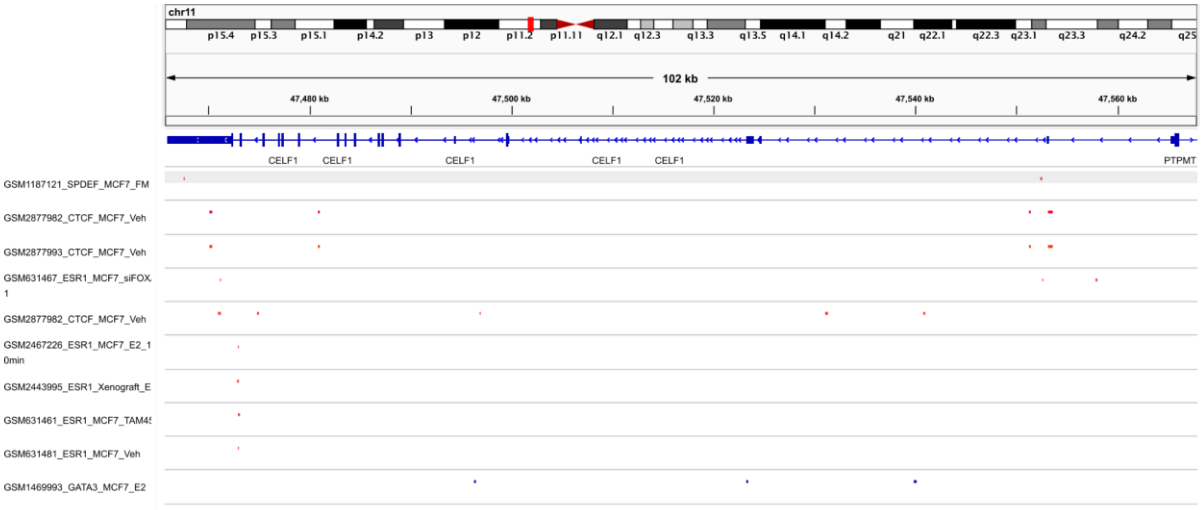

Figure S6. Screenshot of the integrative genome viewer (IGV) reporting ChIP-Seq binding peaks of ERα, SPDEF, CTCF, and GATA3 showing a binding peak at the gene body and 3′UTR region of CELF1 in MCF-7 BC cells in Full Medium (FM), Vehicle (Veh), or under estrogen (E2) treatment. The 3′UTR region of CELF1 is differentially spliced uponERα silencing.

References

1. Shapiro, I.M.; Cheng, A.W.; Flytzanis, N.C.; Balsamo, M.; Condeelis, J.S.; Oktay, M.H.; Burge, C.B.; Gertler, F.B. An EMT-Driven Alternative Splicing Program Occurs in Human Breast Cancer and Modulates Cellular Phenotype. *PLoS Genet.* **2011**, *7*, e1002218.
2. Yang, Y.; Park, J.W.; Bebee, T.W.; Warzecha, C.C.; Guo, Y.; Shang, X.; Xing, Y.; Carstens, R.P. Determination of a Comprehensive Alternative Splicing Regulatory Network and Combinatorial Regulation by Key Factors during the Epithelial-to-Mesenchymal Transition. *Mol. Cell. Biol.* **2016**, *36*, 1704–1719.
